# The Spread of *Mycobacterium chimaera* from Heater–Cooler Units and Infection Risk in Heart Surgery: Lessons from the Global Outbreak?

**DOI:** 10.3390/pathogens13090781

**Published:** 2024-09-10

**Authors:** Anna Maria Spagnolo, Osvalda De Giglio, Giuseppina Caggiano, Francesco D’Agostini, Mariano Martini, Davide Orsini, Sebastiano La Maestra

**Affiliations:** 1 Department of Health Sciences, University of Genova, Via Pastore 1, 16132 Genova, Italy; 2 Interdisciplinary Department of Medicine, Hygiene Section, University of Bari Aldo Moro, Piazza G. Cesare 11, 70124 Bari, Italy; 3 University Museum System of Siena (SIMUS), History of Medicine, University of Siena, 53100 Siena, Italy

**Keywords:** *Mycobacterium chimaera*, infection risk, water contamination, heater–cooler units, whole-genome sequencing

## Abstract

*Mycobacterium chimaera* (MC), a member of the *Mycobacterium avium* complex, can cause infections in patients after open-heart surgery due to contaminated heater–cooler units (HCUs). The transmission route of HCU-related MC infection is non-inhalational, and infection can occur in patients without previously known immune deficiency. Patients may develop endocarditis of the prosthetic valve, infection of the vascular graft, and/or manifestations of disseminated mycobacterial infection (splenomegaly, arthritis, hepatitis, nephritis, myocarditis, etc.). MC infections have serious outcomes (30–50% recurrence rate, 20–67% mortality rate). In 2015, an international outbreak of *M. chimaera* infections among patients undergoing cardiothoracic surgeries was associated with exposure to contaminated LivaNova 3T HCUs (formerly Stöckert 3T heater–cooler system, London, United Kingdom). In response to the global outbreak, many international agencies have issued directives and recommendations in order to reduce the risk of MC infection in cardiac surgery. Whole-genome sequencing (WGS) technology can be used to describe the global spread and dynamics of MC infections, to characterize local outbreaks, and also to identify sources of infection in hospital settings. In order to minimize the risk of contamination of HCUs and reduce the risk of patient infection, it is imperative that healthcare facilities establish a program of regular cleaning and disinfection maintenance procedures as well as monitoring of the water used and the air in the operating room, in accordance with the manufacturer’s procedure.

## 1. Introduction

In the context of clinical risks, infectious risk (the probability of contracting an infection during healthcare facility stays) is the most serious and common adverse effect and/or complication of healthcare that impacts morbidity, mortality, and quality of life [1].

Several pathogens are responsible for healthcare-associated infections (HAI), such as *Staphylococcus aureus*, *Clostridioides difficile*, *Klebsiella pneumoniae*, *Pseudomonas aeruginosa*, *Acinetobacter baumannii*, etc. [2,3,4]. Cases of HAI have also been described by non-tuberculous mycobacteria (NTM) [5,6]. NTM, also known as environmental mycobacteria, atypical mycobacteria, and mycobacteria other than tuberculosis, are mycobacteria which do not cause tuberculosis or leprosy/Hansen’s disease.

To date, more than 180 species have been identified; of these, only a small number can cause infections that mainly affect the lungs, lymph nodes, soft tissues, and skin but is also responsible for infections associated with implants and catheters, as well as surgical wound infections. Species belonging to the *M. avium* complex (MAC) (*M. chimaera*, *M. intracellulare*, and *M. avium*) are the most widespread globally [7].

*M. chimaera* (MC) was initially misidentified as *Mycobacterium intracellulare*. However, it was not until 2004 that Tortoli et al. identified *M. chimaera* as a separate species within the MAC complex [8].

In 2006, during a study in Germany, researchers sequenced the 16–23S internal transcribed spacer region of 166 clinical isolates originally identified as *Mycobacterium intracellulare* by a tertiary hospital and a national reference laboratory for mycobacteria. The study found that 86% of these isolates were *Mycobacterium chimaera* sp. nov. [9,10].

*M. chimaera*, as well as the other MAC group, exhibits a highly lipophilic cell wall, a low number of porins associated with a variety of efflux pumps, and inducible resistance mechanisms, which confer a resistant cell wall and natural drug resistance [11]. It is intrinsically resistant to most classes of antibiotics and disinfectants. Compared to *Escherichia coli*, a bacterium used by disinfection industries as a standard reference, MC is about 1000 times more resistant to chlorine [12].

*M. chimaera* is very common in the environment (water and soil) and can cause infections of varying severity affecting the respiratory tract, soft tissue, and surgical wounds [13]. Garcia-Prieto et al. described an exceptional case of endobronchial fibroanthracosis associated with *Mycobacterium chimaera* infection [14].

This review addresses the worldwide public health issue related to the possible occurrence of serious infections in open-heart surgery patients due to the contamination of heating–cooling units and the main prevention and control measures that should be implemented.

## 2. *Mycobacterium chimaera* Infections and Epidemiological Data

*M. chimaera* was previously reported as an opportunistic respiratory pathogen associated with underlying lung disease, such as chronic obstructive pulmonary disease; it was only later that it was also identified as a causative agent of invasive infections in patients undergoing cardiothoracic surgeries, caused by the contamination of water of heater–cooler units (HCUs) and thermoregulatory components of Extra Corporeal Membrane Oxygenation (ECMO) [15,16], medical devices used during this type of surgery.

Initial symptoms and signs reported in cardiac surgery patients with these infections are unexplained or persistent fever, unexplained weight loss, persistent cough, or shortness of breath, sometimes with blood, asthenia, night sweats, joint or muscle pain, abdominal pain, redness, pain, warmth or pus in the area of the surgical wound, or nausea or vomiting [17]. Patients may develop endocarditis of the prosthetic valve, infection of the vascular graft, and/or manifestations of disseminated mycobacterial infection (splenomegaly, arthritis, hepatitis, nephritis, and myocarditis, etc.) [10].

The transmission route of HCU-related *M. chimaera* infection is non-inhalational, and the likely route of transmission is direct contamination of the open-chest cavity with *M. chimaera*-containing aerosols during cardiac surgery. Infection can occur in patients without previously known immunodeficiency. This is in contrast to pulmonary NTM disease, where NTM-containing aerosols lead to pulmonary infection in patients with significant underlying structural lung disease or who are immunocompromised.

Although the majority of infections have followed open-chest cardiac surgery, infections have also been reported among patients following minimally invasive cardiac surgery [18]. The hypothesized route of exposure among the latter is contamination of surgical equipment or grafts in the operating room by HCU-generated bio-aerosols [19].

Treatment of NTM infections often require combination therapy with multiple classes of antimycobacterial drugs. Macrolides are considered the mainstay of therapy, as with other NTM infections. Other first-line agents include rifamycins, ethambutol, and amikacin. In patients who are not candidates for first-line agents, second-line agents can be used, including clofazimine, linezolid, bedaquiline, and moxifloxacin [20].

MC infections have serious outcomes (30–50% recurrence rate, 20–67% mortality rate) [13].

The first report of *M. chimaera* infection in patients who had undergone open-heart surgery was published by a group of researchers from the University of Zurich [21], who described two cases of *M. chimaera* infection (bloodstream infection and prosthetic valve endocarditis) which occurred in 2011 in two patients who had undergone cardiac surgery (the first patient in 2008, and the second in 2010), with the use of extracorporeal circulation. The authors hypothesized that the source of the infection could be hospital-based; intensive searches were performed in the hospital in 2012, and samples were taken from the faucet water of the operating room, faucet water of the cardiac surgery intensive care unit, condensing water from surgical heater–cooler units (HCUs), hemofiltration solutions, and cardioplegia solutions. Environmental monitoring did not detect an *M. chimaera* strain with a RAPD-PCR pattern identical to that of the isolates from the two case patients. So, a nosocomial link was not identified at the time [20,21].

Since 2011, at least one hundred and eighty cases of *Mycobacterium chimaera* infections, often fatal, have been reported globally, involving five HCU manufacturers and over one hundred facilities [13,22]. However, the real magnitude seems to be critically underestimated.

Cases have been reported in Switzerland, France, Germany, Ireland, Italy, the Netherlands, Spain, and the United Kingdom, as well as the United States, Canada, Australia, and Hong Kong, among patients who had undergone cardiothoracic surgery [22,23]. Afterwards, *M. chimaera* contamination of HCUs has been linked to multiple invasive infections worldwide [15].

In 2015, an international outbreak of *M. chimaera* infections among patients undergoing cardiothoracic surgeries was associated with exposure to contaminated LivaNova 3T heater–cooler units (formerly the Stöckert 3T heater–cooler system, London, United Kingdom). The 3T HCUs were found to disperse *M. chimaera*-containing aerosols from the internal water tanks into the operating room through an exhaust vent, likely leading to patient infection [19,24].

As supported by the results of several epidemiological studies, it is believed that contamination at the LivaNova plant may have been the main source of MC infections in patients previously undergoing open-heart surgery [12].

Italy was also involved in this outbreak, and the first report of a patient infected with MC following cardiac surgery occurred in late June 2018 and was related to a patient who underwent surgery in 2015 [17].

Recently, Cannas et al. [13] reported the results of a retrospective surveillance conducted in Italy, covering the years 2010–2022; 40 possible cases of patients infected with MC were identified, of whom 21 died. Whole-genome sequencing (WGS) was used to analyze 36 strains isolated from these patients, which were found to belong to genotypes 1.1 or 1.8 (outbreak-related genotypes). In addition, *M. chimaera* (genotypes 1.1 or 1.8) was detected in 251 HCUs, and genotypes 1.1 or 1.8 were found in 28 of them.

## 3. The Challenges of Diagnosis of *Mycobacterium chimaera* Infections

The diagnosis of cardiac *M. chimaera* infection can be difficult. This is because initial symptoms may be non-specific, subtle, and appear months to years after surgery. Symptom development occurs, on average, 15–17 months post-surgery, but the incubation period can range from 6 weeks to more than 5 years [19,20].

When a case is suspected or confirmed based on culture or histopathology results, it is necessary to determine whether the infection is localized or disseminated. Mycobacterial cultures of urine, sputum, or fluids/tissues from any organ that might be involved should be under consideration [20].

Based on ECDC guidance [10], the diagnostic criteria of *M. chimaera* infections after cardiothoracic surgery are summarized in Table 1.

**Figure 1:**
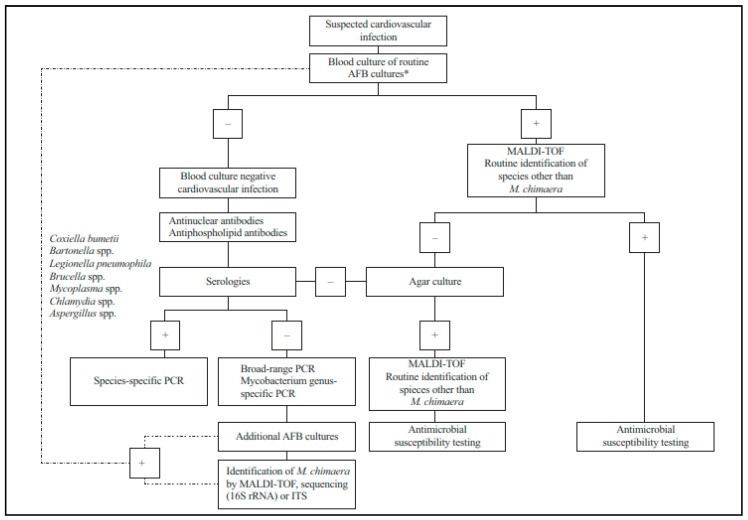
Algorithm for microbiological diagnosis of suspected cardiovascular infections including possible *M. chimaera* infections (reproduced with permission from Hasse B.) [19]. AFB, acid-fast bacilli; MALDI-TOF, matrix-assisted laser desorption–ionization time-of-flight mass spectrometry; PCR, polymerase chain reaction. * Among patients meeting exposure criterion and having symptoms suggestive of *M. chimaera* infection in clinic (assess via upfront AFB cultures).

According to the “International Society of Cardiovascular Infectious Diseases Guidelines for the Diagnosis, Treatment and Prevention of Disseminated *Mycobacterium chimaera* Infection Following Cardiac Surgery with Cardiopulmonary Bypass”, published by Hasse et al. [19], a laboratory diagnostic algorithm for use in cases of suspected *M. chimaera* infection is provided in Figure 1.

## 4. Microbiological Identification and Whole-Genome Sequencing (WGS) in the Management of NTM Outbreaks

MAC species-level differentiation is one of the critical points for the correct diagnosis of NTM infection. It is difficult to distinguish *M. chimaera* from *M. intracellulare* (MI) using the majority of the diagnostic laboratory tests routinely used, because of the great genetic similarity existing between these two species [16]. The complete 16S rDNA gene sequences of MAC species differ by only 6–10 base pairs, and only 1 base pair discriminates *M. chimaera* and *M. intracellulare*. Therefore, sequencing of the 16S–23S internal transcribed spacer region (ITS) has been suggested, although it is rarely available in clinical laboratories [19].

In the last few years, MALDI-TOF mass spectrometry has been widely used to quickly and efficiently identify bacteria, yeasts, and mycobacteria. Effectively, the MALDI-TOF MS system has greatly simplified the identification of mycobacteria; however, at present, the main limiting element seems to be the database of mycobacterial spectra available in the system [25]. To improve this limitation, the company Bruker has developed an enhanced spectral interpretation algorithm in the MALDI-TOF MS system, to better differentiate between the two species, based on differential spectral peaks [16].

There are also several methods for the molecular typing of MAC. However, no standardized or validated subtyping system has been reported for the molecular typing of *M. chimaera* [10].

In recent years, the use of whole-genome sequencing (WGS) techniques has offered the possibility of overcoming the limitations of conventional tests used to diagnose mycobacterial infections, also allowing a better understanding of the global diversity of NTM species. WGS can identify all the single-nucleotide polymorphisms (SNPs) associated with resistance as well as phylogenetic SNPs characteristic of individual NTM species [7,25]. Therefore, the use of this technique has facilitated the characterization of new species of mycobacteria and the identification of gene mutations encoding virulence factors and resistance. WGS data can also be used to describe the global spread and trends of NTM infections, to characterize local outbreaks, and to identify sources of infection in hospital settings.

WGS analysis is a reference method used in molecular epidemiology to investigate the origin of *M. chimaera* infections and to confirm its relatedness to the HCU outbreak strain. Strikingly, the WGS results have confirmed the existence of a common source for the current global epidemic of *M. chimaera*.

Most studies have revealed that the majority of *M. chimaera* patient isolates, HCU water isolates, and air isolates, from multiple countries, were very closely related with differences in single-nucleotide polymorphisms of fewer than 10 variants [19,22].

Therefore, WSG is now an essential component of clinical diagnostics in mycobacteriology laboratory, although it is currently available in only a few reference laboratories to which strains must be sent when clinicians deem it necessary.

## 5. Environmental Microbiology Investigations and Microbial Contamination of Heater–Cooler Units (HCUs)

After the first reports of MC infections in patients undergoing cardiovascular surgery in several cardiothoracic surgery centers across Europe, several environmental microbiology investigations of potential sources of perioperative contamination were undertaken. In the Swiss university hospital where the first outbreak was studied, following a later epidemiologic investigation [26], contamination with *M. chimaera* and other waterborne opportunistic pathogens was recorded in the water tanks and pipes of heating–cooling units used to warm and cool patients’ blood during cardiopulmonary bypasses [10,24].

In addition, *M. chimaera* was isolated from air samples taken in the operating room during surgical procedures and from drinking water samples in the hospital’s fountains. However, biomolecular analysis did not show similar patterns between clinical and environmental isolates [10].

As MC has been isolated in HCUs and also in air samples of the operating room where these devices were used, it is believed that one of the main sources of contamination is the aerosol generated from the water contained in the tanks of the devices, which then spread into the operating room [23].

Therefore, to date, the primary mode of transmission of MC has been identified in the water tanks of HCUs, which produce a contaminated aerosol. Stagnation of water and high temperatures (up to 40 °C) promote the formation of biofilms, creating a more favorable environment for *M. chimaera* [11]. Indeed, the aggregation of microorganisms in the form of biofilms on surfaces in contact with water increases their ability to survive and, under favorable conditions, to multiply. Rich in nutrients and having a protective effect on microorganisms against disinfectants, it is also a potential site for the transfer of virulence and antibiotic resistance traits. In its structure, where organisms are trapped in a matrix of various kinds, extracellular polymeric substances (ESPs) mainly composed of polysaccharides and proteins predominate, forming highly hydrated matrices.

Observations by 3D laser scanning microscopy has shown that MC has motility during surface colonization and can form biofilms on stainless steel, titanium, silicone, and polystyrene surfaces during the first week of inoculation. Scanning electron microscopy (SEM) of *M. chimaera* biofilms after 4 weeks of inoculation has shown that MC cells are entirely enclosed in extracellular material [15].

In the case of HCUs, MC contamination can occur at any time (at the production site, during machine preparation before an operation, while the machine is stationed in the hospital, between operations, etc.) [23].

In a study conducted by van Ingen et al. [22], 250 isolates (clinical and environmental) of *M. chimaera* underwent phylogenetic analysis based on WGS. Of these, 24 were clinical isolates from patients undergoing cardiac surgery in different countries: Switzerland, Germany, the Netherlands, and the United Kingdom. The significant clonality of the isolates from almost all patients with *M. chimaera* infections strongly indicated a common source of infection. The high degree of similarity between isolates recovered from most LivaNova HCUs in clinical use and from the LivaNova manufacturing site and patient isolates suggested that the LivaNova HCUs represented the common source of infection and that contamination of most of these HCUs presumably occurred during production at the manufacturing site.

Bisognin et al. [27] analyzed 417 water samples from hospitals located in three Italian provinces; the samples were collected from 52 HCUs (Stockert 3T (n = 41); HCU40 (n = 11)) and from 23 hospital faucets used to fill the HCUs’ tanks. The samples were concentrated and cultured for MC. The positive cultures (n = 53) were characterized by IR-Biotyper system and WGS analysis. Three separate clusters of MC attributable to each hospital were determined. The one positive MC sample from tap water clustered with isolates from the HCUs of the same hospital, supporting that plumbing water could be the source of contamination of the HCUs and, potentially, infection of patients.

Tuvo et al. [28] analyzed 82 water samples collected from 24 HCUs: 3T LivaNova (n = 11), 1T LivaNova (n = 3), TCM-Sarns Terumo (n = 1), and HCU40 Maquet (n = 9). *M. chimaera* was detected in 12/82 (15%) (strain CP015272.1) of the samples.

Similarly, Nicoletti et al. [29], in the period 2017–2022, collected 1191 samples from 35 HCUs, of which 16 were from the 3T HCUs of LivaNova company (type 1), 2 were from the HCU40, and 17 were from the HU35 both of Maquet (type 2). Overall, 118 positive for *M. chimaera* (10.3%) were identified; specifically, 83 in the HCUs were type 1 (21.6%) and 35 were type 2 (4.6%).

Water contained in HCUs can also be contaminated with other potentially dangerous microorganisms. In a study by Garvey et al. [30], water samples were taken from four HCUs. Water counts yielded a total viable count >300 CFU/100 mL, with Gram-negative bacteria (Coliforms, *Pseudomonas* spp., etc.), atypical mycobacteria, and fungi. The HCUs were opened up, and visible biofilm was identified in all of the tubing within the unit. The manufacturer’s guidance for decontamination of HCUs was modified in this study; specifically, the new decontamination program included initial mechanical removal of biofilm, replacement of parts of the HCUs, and two consecutive cycles of peracetic acid disinfection. After this initial decontamination, maintenance was intensified with daily water changes with filtered tap water and addition of hydrogen peroxide to the HCU tanks. In addition, weekly decontamination of the entire system with peracetic acid was performed. Although water sampling produced no microorganisms following this decontamination regimen, previous reports have suggested that subsequent contamination is still possible.

## 6. Prevention and Control Measures Adopted in Europe

In response to the outbreak, many international agencies, including the Centers for Disease Control and Prevention (CDC), Food and Drug Administration (FDA), and the European Centre for Disease Prevention and Control (ECDC) have issued directives and recommendations in order to reduce the risk of MC infection in cardiac surgery.

In 2015, a special task force was formed within the competent authorities’ expert surveillance group, established at the EU Commission, which investigated the issue in depth and collaborated with other member states in order to identify the most appropriate corrective actions [23]. The group investigated and retrieved all available data, which were unfortunately few due to the lack of specific surveillance protocols. Nevertheless, the data provided an initial epidemiological description of the problem, and initial operational actions were introduced [12].

Also in 2015, ECDC disseminated a Rapid Risk Assessment [31] throughout Europe to all healthcare personnel for infections associated with this type of equipment. At the end of 2016, another European ECDC Rapid Risk Assessment was issued with new preventive measures such as the placement of HCUs outside operating rooms [12,23]. ECDC also published, in August 2015, the “EU protocol for case detection, laboratory diagnosis and environmental testing of *M. chimaera* infections potentially associated with heater–cooler units” [10].

In addition, the Health Security Committee, composed of experts from the European Commission, has issued a statement to facilitate the exchange of information among Member States, to implement appropriate control measures throughout Europe, and to coordinate European authorities when new cases are identified [23].

Since 2014, the company LivaNova has issued several Safety Alerts and Recommendations, indicating the serial numbers of affected products and the procedures to be taken [17]. Moreover, in October 2018, the 3T HCUs’ manufacturer released updated disinfection protocols and introduced device upgrades to reduce the risk of airborne transmission of NTM [24].

In 2019, Italy issued national recommendations for the control of *M. chimaera* infection [17] and operational guidance regarding the diagnosis and laboratory identification of *M. chimaera* [32] and regarding follow-up and notification aspects of infection cases [33], improving the ability to detect cases and contaminated devices.

In order to minimize the risk of contamination of HCUs and reduce the risk of patient infection, it is imperative that healthcare facilities establish a program of regular cleaning and disinfection maintenance procedures as well as monitoring of the water used and the air in the operating room, in accordance with the manufacturer’s procedures.

If these procedures for HCUs are not carried out scrupulously, there is a possibility that microorganisms can survive inside the water tanks, creating a biofilm, which provides a suitable environment for bacteria, including mycobacteria, to proliferate, resulting in the possibility of spreading in the form of aerosols when the device is operating. In addition, as a precautionary measure, HCUs should be placed as far away as possible from the surgical site or, in any case, positioned to direct the outlet flow of the fans away from the surgical site and near the aspiration system of the operating room [23]. Table 2 shows interventions for reducing the risk of infection, based on the 2019 national recommendations [17].

Most recently, the International Society for Cardiovascular Infectious Diseases (ISCVID) recognized the growing need for international guidance on diagnosis, management, and prevention of these infections. The primary aims of guidance published in 2020 [19] were to provide an update on MC epidemiology and risk factors, develop guidelines for diagnosis and management in individual patients, and outline infection prevention and control recommendations.

## 7. Conclusions

Given the peculiarity of these infections, which occur at a considerable distance in time, the possibility of retrospectively identifying exposed patients is crucial. Of considerable importance is the setting up of a registry at cardiac surgeries and other relevant wards, with prospective value, to track the cooling/heating devices used in patients undergoing open-chest surgery and the information of general practitioners and hospital and territorial specialists on the potential risk of *M. chimaera* infections following open-chest surgery.

In addition, it is important that centers using HCUs follow the decontamination instructions provided by the manufacturers and the various national and international recommendations for preventing contamination of HCUs.

Also of great importance is the periodic monitoring of HCUs tank water, tap water, and operating room air in order to identify the presence of *M. chimaera* early on, enabling the prevention of its environmental spread.

Further research is needed on many aspects of diagnosis, management, and prevention, as well as on the development of decontamination procedures that are more efficient in disrupting biofilms; in addition, an improvement in the design of HCUs for facilitating decontamination is also advisable.

## Figures and Tables

**Table 1:** *Mycobacterium chimaera* case definition (modified from [10]).

Clinical Criteria Any of the Following:	Exposure Criteria	Probable Case	Confirmed Case
- Prosthetic valve endocarditis - Prosthetic vascular graft infection - Sternotomy wound infection - Mediastinitis - Manifestations of disseminated infection including embolic and immunologic manifestations, e.g., splenomegaly, nephritis, myocarditis, arthritis, osteomyelitis, bone marrow involvement with cytopenia, chorioretinitis, lung involvement, hepatitis.	Surgery requiring cardiopulmonary bypass in the five years prior to the onset of symptoms of infection.	A patient meeting the clinical and exposure criteria **AND** *M. chimaera* detected by direct PCR and amplified DNA sequencing from an invasive sample (blood, pus, tissue biopsy or implanted prosthetic material). **OR** MAC detected by culture or direct PCR from an invasive sample (blood, pus, tissue biopsy or implanted prosthetic material). **OR** Histopathological detection of non-caseating granuloma and foamy/swollen macrophages with acid fast bacilli in cardiac or vascular tissue in the proximity of the prosthetic material or in specimen from the sternotomy wound.	A patient meeting the clinical and exposure criteria. **AND** *M. chimaera* detected by culture and identified by DNA sequencing in an invasive sample (blood, pus, tissue biopsy or implanted prosthetic material).

MAC = *Mycobacterium avium* complex; PCR = polymerase chain reaction.

**Table 2:** Italian recommendations for the prevention of *Mycobacterium chimaera* infections [17].

(a) In order to minimize the risk of infection of the patient undergoing cardiac surgery using HCUs, All cardiac surgical departments must conduct a risk assessment in relation to the HCUs in use, taking into consideration the following parameters: manufacturing company/model/matriculation/year of manufacture, performance of retrofit interventions aimed at reducing aerosol risk, type/periodicity of disinfection procedures, and outcomes of microbiological monitoring on water and possibly air. Based on this assessment, consequent interventions should be activated. HCUs that have been identified as contaminated should be excluded from use until they are cleaned. All HCUs, from any manufacturer and in all hospital facilities, precautionarily, should be appropriately placed relative to the operating bed, according to the guidance provided in published Ministerial Safety Notices. If feasible, the device should be placed outside the operating room. If not feasible, and considering the characteristics of the operating room available, the discharge of the heating–cooling device should be directed/channelized away from the patient, e.g., toward the operating room’s exhaust vent. Strictly observe the instructions for use, especially those related to cleaning and disinfection. Carry out water quality monitoring. Establish, at the level of the individual care facility, a procedure for tracking the HCU device used for each cardiac surgery, so that patients possibly at risk of infection, who are exposed to a device found to be contaminated on testing, can be easily identified retrospectively.
(b) HCUs related to one or more cases of *M. chimaera* infection should be suspended from use until the results of the microbiological investigations carried out are available, which should include searching for biofilm and any deposits within the reservoirs: In case of a negative result, the device may be returned to service, following the instructions given in point a. In case of a positive result, the device will require the appropriate decontamination procedures, and the manufacturer’s instructions should be scrupulously followed. The device may be used only after ascertaining the absence of risk.
(c) After identification of a contaminated HCU and suspension of its use, if a different HCU is used, new fittings, tubing, and connectors should be used to avoid contamination of the new equipment.

## Data Availability

Data sharing is not applicable.
